# Zebrafish Avatars towards Personalized Medicine—A Comparative Review between Avatar Models

**DOI:** 10.3390/cells9020293

**Published:** 2020-01-25

**Authors:** Bruna Costa, Marta F. Estrada, Raquel V. Mendes, Rita Fior

**Affiliations:** Champalimaud Centre for the Unknown, Champalimaud Foundation, 1400-038 Lisbon, Portugal; bruna.costa@research.fchampalimaud.org (B.C.); marta.estrada@research.fchampalimaud.org (M.F.E.); raquel.mendes@research.fchampalimaud.org (R.V.M.)

**Keywords:** cancer, zebrafish, Avatars, tumor microenvironment, PDX, personalized medicine

## Abstract

Cancer frequency and prevalence have been increasing in the past decades, with devastating impacts on patients and their families. Despite the great advances in targeted approaches, there is still a lack of methods to predict individual patient responses, and therefore treatments are tailored according to average response rates. “Omics” approaches are used for patient stratification and choice of therapeutic options towards a more precise medicine. These methods, however, do not consider all genetic and non-genetic dynamic interactions that occur upon drug treatment. Therefore, the need to directly challenge patient cells in a personalized manner remains. The present review addresses the state of the art of patient-derived in vitro and in vivo models, from organoids to mouse and zebrafish Avatars. The predictive power of each model based on the retrospective correlation with the patient clinical outcome will be considered. Finally, the review is focused on the emerging zebrafish Avatars and their unique characteristics allowing a fast analysis of local and systemic effects of drug treatments at the single-cell level. We also address the technical challenges that the field has yet to overcome.

## 1. The Problem: “One Size” Does Not Fit All

The arrival of precision medicine and immunotherapy are giving hope to finally manage cancer treatment. Many advances were made possible due to the discovery of molecular pathways in tumor cells and on their interaction with the surrounding tumor microenvironment (TME) [1], leading to the development of many new therapies. The latest successes in HER2-targeted therapy and the discovery of immune checkpoint inhibitors are great examples of this [2]. However, not all patients respond and many are not eligible for these “new therapies”. Thus, nowadays, the majority of patients are still treated with traditional chemo/radiotherapy and surgery according to clinical guidelines, which are developed and approved based on average efficacy rates.

As a result of this “one-size-fits-all” approach, treatments may prove to be efficient for some patients but not for others. For example, in the international therapeutic guidelines (NCCN and ESMO) for advanced colorectal cancer (CRC) there are two main chemotherapeutic arms (FOLFOX and FOLFIRI), which show very similar response rates of ~50% [3,4]. In other words, ~50% of patients respond to treatment while ~50% do not. Clinicians do not know in which group patients will fall, as there is no predictive screening test to provide this information. Thus, patients that start with FOLFOX and do not respond change to FOLFIRI, and vice-versa. This applies for CRC and many other cancers, being especially relevant in the metastatic scenario when oncology therapy guidelines reach branch points and clinicians face equivalent options. At this point, many patients go through a trial-and-error type of approaches (according to the guidelines), being exposed to unnecessary treatments with severe side effects and extremely high healthcare costs. This poses the question: how to choose the best treatment for each individual patient?

## 2. Moving Away from the “One-Size-Fits-All Approach” towards Precision and Personalized Medicine

### 2.1. Characterizing the Tumor–Patient Stratification

Recent genome cancer profiling studies have revealed the huge genetic heterogeneity of cancer. This heterogeneity has been observed not only between cancers (inter-tumor/inter-metastatic/inter-patient), but also within each cancer (intra-patient) [5,6]. Genetic diversity impacts on multiple tumorigenic phenotypes, such as activation of specific signaling pathways, senescence, secretion of soluble factors, migratory and invasive capacity, activation of metabolic pathways, ability to metastasize, and ultimately on the response/resistance to treatment [6]. Even identical CRC cells that share the same genome may exhibit multiple functional profiles (including distinct responses to therapies), implying that the basis for heterogeneity can be not only genetic but also epigenetic or/and environmental [7].

Diversity poses a major challenge for drug development and precision medicine. This is why there is a major effort to stratify patients to be able to pinpoint the best therapy for each individual patient. Nowadays, in most hospitals and as a routine, this classification is mostly based on the histological and molecular profiling of tumors, allowing the distribution of patients through different subtypes within a cancer. More specifically, tumor subtypes can be defined based on: the level of differentiation of a tumor; the expression of specific molecules such as the hormone receptors in breast or prostate cancer; mutation burden; microsatellite instability; stromal or immune cell content; and extracellular matrix composition; amongst many others [8,9,10,11,12].

### 2.2. Pharmacogenomics: “Tell Me Your Genes and I Will Give You Your Drug”

In an attempt to predict patient’s response to specific drug regimens, tumors are being characterized at the molecular level [13]. Pharmacogenomic studies focus mainly on the genomic and transcriptomic profiling and their association with drug sensitivity or resistance, leading to the development of many biomarkers that contribute to a better stratification of patients. One example is the treatment of lung-cancer patients positive for Epidermal Growth Factor Receptor (EGFR) mutations, which are now treated with EGFR tyrosine kinase inhibitors as first line of therapy [14]. Another example are breast cancer patients with high Estrogen Receptor (ER) expression that receive endocrine therapies like ER antagonists or aromatase inhibitors [15,16], or the HER2+ breast and stomach cancers that can be treated with anti-HER2 antibodies [17,18].

Although these therapies provide an outstanding improvement in survival, only ~55% of HER2-positive patients show a pathological complete response [17]. In other words, even in outstanding therapies with robust biomarkers, not all patients respond. This is likely due to the tumor’s heterogeneity, which may present HER2 negative clones that are not dependent on HER2 signaling and, therefore, become dominant after treatment. Other possibilities are the presence of other mutations that interact in complex signaling networks, or signaling/metabolic rewiring that might occur as an adaptation to treatment [19,20].

Thus, pharmacogenomics, proteomics and metabolomics technologies capture these “*omic*” static states of the initial cancer. These methods, however, do not take into account all the possible genetic interactions that may occur, between different subclones or with the TME. These are a “frozen picture” of dead cancer cells, which lack quantification of their function and response to direct perturbation.

## 3. Challenging Directly Tumor Cells—“Test Thy Cells”

### In Vitro Chemosensitivity Tests

**Figure 1:**
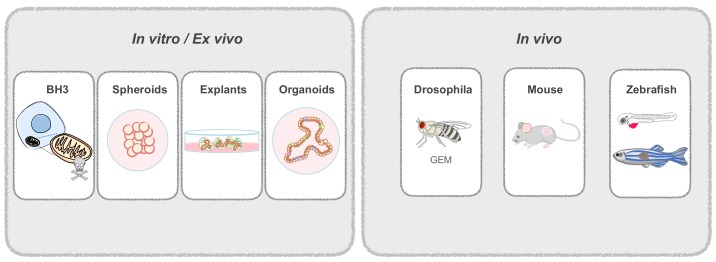
Patient-derived Avatars. Patient-derived cells are used to generate in vitro and in vivo Avatars. In vitro models include spheroids from dissociated tissue; explants, that are not dissociated and retain the original tissue architecture; and organoids, derived from adult stem cells. In vivo models include genetically engineered drosophila flies that mimic patient mutations; and patient-derived mouse or zebrafish xenografts. Zebrafish can be used at the larval or adult stage.

Several in vitro cell culture chemotherapy sensitive and resistant assays (CSRAs) have been developed to fulfil the needs to predict the response to a given treatment, yet with very limited success. In these tests, patient tumor cells are placed in culture for 3–14 days and exposed to therapies, after which cell death, proliferation or colony formation are evaluated [21]. Due to the lack of predictive power, these assays are not recommended in oncology practice [21]. This is probably due to some limitations of the 2D assays, such as relying only on indirect viability assays (ATP or MTT or EDRA or ChemoFx) [21] and/ or the absence of a 3D architecture or a tumor microenvironment. Moreover, these in vitro assays rely on several passages in culture for amplification, subjecting cells to a strong selection pressure. Nevertheless, exciting new methods are emerging, some of which we address in this review (Figure 1).

## 4. What Is New in the Last Years?

### Dynamic BH3 Profiling

Dynamic BH3 profiling (DBP) relies on evaluating the apoptotic sensitivity of tumor cells, or in other words: “How much are cells primed to die?” The more “primed” cells are, the more sensitive to chemotherapeutic agents they may be, making this quick assay a possible predictor of chemosensitivity in vitro. This assay works as a readout of the balance between pro- and anti-apoptotic proteins, which will activate or block the apoptotic cascade leading to cell death [22].

**Figure 2:**
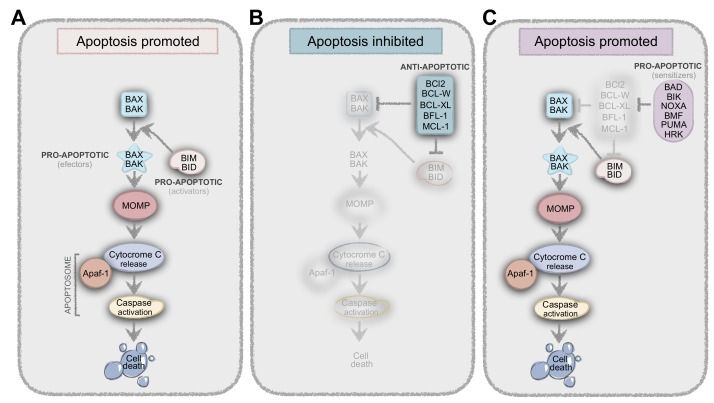
Balance between pro- and anti-apoptotic proteins result in life-death decisions. (**A**) BIM and BID (activators) are known as the BH3-only proteins whose function is to induce the conformational changes of BAX and BAK (effectors) to produce the Mitochondrial Outer Membrane Permeabilization (MOMP), resulting in apoptosis. (**B**) Anti-apoptotic proteins like BCL-2, BCL-W, BCL-XL, BFL-1, and MCL-1, can bind to BH3-only proteins to prevent their interaction with BAX and BAK, thus preventing apoptosis. (**C**) Another class of proteins, which are pro-apoptotic or sensitizers (BAD, BIK, NOXA, HRK, BMF, and PUMA) cannot directly bind to BAX and BAK, but instead bind to anti-apoptotic proteins, preventing the interaction between these and the activators.

Most chemotherapy agents induce cell apoptosis by changing the levels and interactions of the proteins from the BCL2 family. BAX and BAK (effectors) are pro-apoptotic proteins that undergo conformational alterations upon activation, leading to Mitochondrial Outer Membrane Permeabilization (MOMP), cytochrome-c release and, consequently, apoptosis (Figure 2). BIM and BID (activators) are known as the BH3-only proteins whose function is to induce the conformational changes of BAX and BAK to produce the MOMP. However, many anti-apoptotic proteins can bind to BH3-only proteins to prevent their interaction with BAX and BAK and thus prevent apoptosis. On the other hand, pro-apoptotic or sensitizers (like BAD) bind to anti-apoptotic proteins, preventing the interaction between these and the activators (Figure 2). In summary, it is the balance and interaction between all these players (and many others…) that result in life-death decisions (Figure 2) [22,23].

Importantly, by using DBP, Letai and colleagues [22] explain why BCL-2 gene/proteins levels do not correlate with chemo-sensitivity levels in leukemia patients, for instance. They showed that, although BCL-2 levels were high, cells were still sensitive to insults and primed to die, since sensitizers were pre-bound to anti-apoptotic proteins, resulting in a reduction of available anti-apoptotic proteins to bind activators [22,23]. This example illustrates why having the “*omics*” profile might not be enough to predict the therapy outcome, as protein–protein interactions that might be occurring in those cells are not being evaluated.

In this quick test, tumor single-cell suspensions without previous expansion are exposed to selected drugs for 16 hours, after which BH3 priming is quantified. By using this method, the authors were able to demonstrate an impressive correlation between priming percentage and cell death. This also explains why cytotoxic drugs can induce cell death even when cells are not actively proliferating.

Retrospective correlations matched the results obtained from DBP with chronic lymphocytic leukemia (CLL), acute lymphocytic leukemia (AML) and ovarian cancer patient clinical outcomes, with a good correlation [22,23,24].

Although very promising, the extension of this model to other solid tumors and additional retrospective studies are needed in order to validate this approach for other clinical applications.

## 5. Tumors Are Not 2D and Tumor Cells Are Not Alone—The Tumor Microenvironment

In the last decades, many other assays have been developed to culture in vitro patient-derived cells. Most of these models are now developed in 3D, as 2D cultures do not mimic the tridimensionality of living cells and tissues. 3D models allow reconstruction of cell-cell and cell-extracellular matrix (ECM) interactions needed for cell polarization, differentiation, and migration [25,26,27,28]. Moreover, tumor cells are not alone: the tumor ecosystem is comprised by a complex network of fibroblasts, adipocytes, endothelial cells, pericytes, and a variety of immune cells embedded in the ECM [1]. Altogether, these components interact with epithelial cells and directly influence tumor cell behavior [29,30] and therapy sensitivity [31]. Therefore, in an attempt to mimic some aspects of this complexity, many 3D patient-derived in vitro models are currently being developed.

### 5.1. In Vitro Patient-Derived Tumor Models

#### 5.1.1. Spheroids

To generate patient-derived spheroids, tumor pieces of freshly collected tissues are dissociated (partially or completely) and then cultured either in low adhesion plates or embedded in matrigel [32,33,34,35]. In a very recent study with ovarian cancer, spheroids from 92 patients were generated in 24 h and then treated for 72 h with first- and second-line therapies. By comparing patient clinical responses with the spheroid tests, the authors show an outstanding prediction efficiency of 89% [34]. However, despite being 3D, most spheroids lack the original architecture, as well as stromal and immune components, which are crucial modulators of drug response.

#### 5.1.2. Explants and Tissue Slices

Alternatively, patient-derived explant cultures of freshly isolated tumor samples are a physiologically relevant culture system to study the interactions between tumor cells and the surrounding microenvironment [36]. These include tissue slices and explant cultures, where the tissue is cut in pieces, maintaining the original architecture and heterogeneity [35,37].

While this method seems very appealing, explant cultures are very difficult to maintain due to loss of cellularity after 7 days [38,39]. Moreover, we could only find in the literature one study showing retrospective correlation with patient clinical outcome. In this study, the authors performed explant cultures of colon and head and neck squamous cell carcinomas that, upon culture with a matrix-specific scaffold adapted for each tumor, showed a great correlation with the patient’s response [37,40]. Despite being a very promising tool to perform drug sensitivity screens, these models still need validation by other labs and on other solid tumors, in order to be considered for further applications in co-clinical trials.

#### 5.1.3. Organoids

Organoid cultures were a major breakthrough in the in vitro culture of tumor cells from patients, becoming the most attractive tool to be used as an in vitro screening platform.

By definition, organoids are 3D cultures derived from adult stem cells, in which a defined cocktail of soluble factors together with matrigel allow their selection, expansion and differentiation into epithelial structures [41]. More recently, this technique was further developed to generate organoids from patient-derived cancer tissues. These include gastrointestinal, pancreatic, colorectal, hepatocellular carcinoma, cholangiocarcinoma, Barrett’s esophagus, prostate, bladder, breast glioblastoma [42], and ovarian cancer [43], among others. In general, tumor histology was shown to be maintained in all tumor types and subtypes when compared with the parental tumor, including the expression of nuclear hormone receptors in breast and prostate cancer [44,45], although a slight decrease in ER was reported [44]. Moreover, it has been shown that organoids maintain the overall genetic characteristics of the original tissue, including copy number alterations and mutation burden [44,46,47]. For example, results in breast cancer showed that organoids with high BRCA1/2 signature responded to PARP inhibitors treatments, whereas organoids with low BRCA1/2 signatures did not [44].

Although many studies have been published with tumor organoids, to the best of our knowledge, only a few performed retrospective studies where patient clinical outcomes were directly correlated with organoid drug response. There are 3 reports showing a correlation: 2 patients for breast cancer, 4 for gastrointestinal cancer, and 12 patients for colorectal cancer could be effectively matched to the organoid Avatar result [44,48,49]. Another drawback is the time required for the initial organoid generation—4–5 weeks, which is border line for the time frame needed to guide first clinical decisions [41,46]. Also, the lack of stromal components has been a limitation of the organoid culture system that is now being tackled by several strategies. For example, recently, Neal J. et al. [50] have developed an alternative method for organoid generation, where tumor epithelium was propagated together with immune cells, allowing anti-PD1 screening.

Nevertheless, these models still lack many complex interactions observed in the TME or in a living organism and do not allow, for instance, the evaluation of the metastatic or angiogenic potential.

## 6. In Vivo Models—The Complexity of a Living Organism with Patient-Derived Xenografts

Patient-Derived Xenografts (PDXs), also called cancer “Avatars” [51], are generated by the implantation of human primary tumor cells or tissues, obtained from surgery or biopsy, into a host animal. Mouse PDXs are the most widely used but zebrafish PDXs are also emerging as a cheaper, but most importantly, a much faster model.

In vivo models represent a step forward in modeling tumor complexity, as cells are implanted in a living organism where many types of dynamic interactions may occur. This represents a clear contrast with in vitro models where interactions are restricted to the cells already present in the tumor or are artificially reconstructed and maintained by adding high amounts (many times not physiological) of cytokines, growth factors, serum, nutrients, etc.

In a living organism, with all functional organs, with a beating heart, blood and lymphatic systems, liver, bone marrow, kidneys, CNS, etc., tumors can engage in both local and systemic cell-cell interactions, shaping tumor progression. These interactions occur between the tumor and the host and vice-versa, with long distance communication, allowing the recapitulation of cancer hallmarks like cell migration, invasion, metastization, angiogenesis, and immune evasion that are not possible to observe in vitro [52].

When tumor cells are implanted, many different cells from the host are recruited to the tumor site following the instructions of the tumor. These include endothelial cells to reconstruct blood and lymphatic systems, pericytes that cover endothelial cells, tumor-associated macrophages (TAMs), cancer-associated fibroblasts (CAFs) and other immune cells that constitute the complex TME [1,53,54]. These recruited cells can either be tissue-resident cells or cells that are summoned through systemic recruitment, many times coming from distant organs like the bone marrow [55,56].

Several examples illustrate this dynamic cross-talk between cells: macrophages facilitate tumor cell intravasation into the bloodstream, enabling metastasis [57,58]; the preparation of pre-metastatic niches by bone marrow-derived cells, previously “educated” by tumor cells [59]; or for instance the host–microbiome interactions, which were recently shown to modulate drug sensitivity/resistance [60].

Moreover, recruitment of all these cells results in alterations of the ECM like increased deposition of collagens or fibronectin and matrix remodeling enzymes. Together, these physical alterations increase matrix stiffness/density [61,62,63] and lead to a high interstitial fluid pressure, modulating the formation of gradients of signaling molecules and drug diffusion/delivery [64], ultimately impacting on tumorigenesis [65,66].

Finally, drug sensitivity profiling of tumor cells using in vivo models allows for the evaluation of pharmacokinetics, pharmacodynamics and toxicity in a whole living organism. In vivo screens have major advantages in relation to in vitro assays, since to produce in vivo phenotypes, compounds must be absorbed, reach targets, circumvent elimination, and cannot be too toxic, otherwise the animal will not survive [67]. The reconstitution of all these and other variables in a tumor-dependent manner makes in vivo models more complex and physiological, and therefore more representative of these tumors.

In summary, complexity of in vitro models is provided according to the expertise of the researcher, whereas in in vivo models, complexity is built according to the instructions and dynamic cues of the tumor itself.

### 6.1. Mouse Patient-Derived Xenografts

Amongst the large repertoire of in vivo systems used to study cancer, mouse Patient-Derived Xenografts (mPDX) represent the most widely used system, being considered the gold standard in transplantation of human tumors and oncology research [68]. With the discovery of nude mice (lacking functional T cells) [69], the first successful transplantation of human tumors was reported in 1969 [70]. Nude mice still have B-cells and NK-cells responses and therefore, in the last decades, increasingly immunocompromised mouse strains were engineered to achieve higher engraftment rates (Rag1/2 null [71,72], NOD/SCID [73], NOG [74]). The need for an immune-compromised animal, in which many components of the TME are absent, is one of the major limitations of mPDXs. To address this, “humanized-xenograft” models are being explored, consisting in the implantation of patient tumor cells together with human peripheral blood mononuclear cells or hematopoietic stem cells. This allows tumor-immune interactions and assessment of immune checkpoint inhibitors such as nivolumab [75,76].

**Figure 3:**
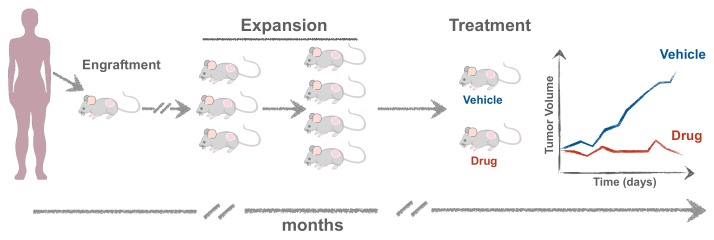
Experimental setup for generating mouse Patient-Derived Xenografts (mPDXs). The tumor is minced and transplanted either orthotopically or subcutaneously, embedded in matrigel. When the tumor reaches ~1 cm in diameter, it is excised and propagated into more mice (F2, F3) to obtain cohorts of Avatars where different therapies can be tested.

The protocol for generating mPDXs is quite simple: the tumor fragment or biopsy is minced and transplanted embedded in matrigel [77] (Figure 3). When patient-derived cells are first expanded in vitro or transformed into cell lines, these are designed Cell-Derived-Xenografts (CDXs). Usually the transplantation is done subcutaneously, since this site facilitates engraftment, monitoring and resection of the tumor. However, mPDXs can also be established orthotopically to the equivalent organ of origin, which may contribute to a more reliable mimicry of the microenvironment [75]. Moreover, within each organ, the site of injection may also differ. For example, breast cancer xenografts are often injected orthotopically in the fat pad of mammary glands. However, injection in the mammary ducts was shown to be more recapitulative of the original tumor phenotype [78,79].

Tumor growth can generally be detected between 1 (sometime less, but rare) and 10 months (occasionally up to 18 months). When it reaches 1 cm in diameter, the tumor is excised and expanded into more animals (>10 mice) [77], to obtain different cohorts of Avatars, in which different therapies can be tested and compared (Figure 3).

#### 6.1.1. Maintenance of Histopathological and Genetic Characteristics

Genomic studies have shown that mPDX models generally maintain the genetic heterogeneity of the original tumors, retaining histopathological characteristics, thus reflecting the uniqueness of each patient [20,68]. Analysis of gene expression profiles shows no substantial changes between donor tumors and their corresponding mPDXs. Particularly, characteristics such as tissue structure, mucin production or cyst development are also maintained in mPDX models [20]. There are some reports, however, showing that, after serial passages, mPDXs can diverge from the original tumor, including in chromosomal ploidy and histology [77].

#### 6.1.2. Correlation of Drug Response with Genetic Signatures and Average Clinical Data

A noteworthy high correlation in drug response between mPDX models and clinical data has been reported, mainly with patient genetic signatures or with reported average responses in groups of patients. In 2011, Bertotti et al. reported the efficacy of cetuximab (anti-EGFR antibody) using 85 PDXs derived from CRC [80]. The frequency of tumor regression, disease stabilization, and disease progression after cetuximab treatment was in line with the average clinical data reported in humans. Finally, identically to clinical observations, KRAS (codons 12 and 13) mutant xenografts were all resistant to EGFR blockade [80].

In 2015, Gao et al. performed a high-throughput in vivo drug screening method using more than 1000 diverse tumor mouse xenografts. The study, also called the *‘PDX Encyclopaedia’* revealed the fidelity of xenografts in confirming the relationship between multiple genotypes and drug sensitivities [81]. By correlating genomic information with observed efficacy, the authors successfully validated genetic hypotheses and biomarkers.

Besides drug efficacy studies, mPDXs can be used for drug discovery, development of new drug combinations, biomarker studies as well as discovery of resistance mechanisms [82,83,84,85,86,87,88].

#### 6.1.3. Correlation of Drug Response with Matched Patient Treatment Outcome

Within the scope of personalized medicine, the implementation of mouse Avatars aims to identify the best therapeutic strategy for each individual cancer patient. To this end, the model had to be validated with retrospective studies to test its predictive value [89,90,91,92,93]. In this scenario, the mouse Avatar is treated with the same therapy as the patient, and the patient response to treatment is compared with its mPDX. For example, Izumchenko et al. [90] compared the patient clinical response with their matching mouse Avatar for several cancer types (sarcoma, breast, ovarian, lung, colorectal, pancreatic, etc.). A significant association was observed in 91 of 129 (71%) therapeutic tests, as tumor growth regression in mPDXs accurately paralleled clinical response in patients [90].

Although still few, some fundamental studies in mice were performed in a prospective manner to guide clinical treatment decisions [76,94,95,96,97]. In 2014, Stebbing et al. [95] established 16 mPDXs from 29 patients with advanced sarcoma. In total, 6 of the patients benefited from mPDX-guided therapy. In the same year, Garralda et al. [94] combined next-generation sequencing with mPDXs to guide personalized treatments for 13 patients with advanced solid tumors. Despite limitations in efficiency, speed and cost, Avatars proved to be useful at tailoring therapy in 5 patients [95]. More recently, Mahecha and colleagues established a mPDX model from a metastatic HER2^+^ gastric cancer patient and tested ado-trastuzumab emtansine as an alternative therapy for the patient, who responded to treatment before relapsing 6 months later [97]. Results from mouse Avatars generally take months to be available. Consequently, most of these studies focus on metastatic stages to specify second lines of therapy, treatments after all other care has been exhausted, or if a therapy does not exist. An exception was the study of Vargas et al. [76], which was able to predict response to first-line therapy (gemcitabine/nivolumab), development of resistance and response to second-line therapy (paclitaxel/neratinib) before these events were observed in the patient. The authors established a mPDX from a patient with metastatic clear cell adenocarcinoma of müllerian origin and developed a co-clinical experimental design to effectively guide patient treatment. This prospective study for first line treatment was only feasible due to the possibility to harvest the tumor within 2 weeks of implantation (although only 5.3% implanted successfully). As pointed by the authors, this was only possible due to the availability of a large amount of tissue from the surgery and its intrinsic rapid proliferation, allowing the generation of multiple mPDXs [76].

In summary, the mouse Avatar is a fundamental model for academic, pharmaceutical and clinical oncology research. Some initiatives for creating and implementing shared large-scale mPDX platforms already exist, including the US National Cancer Institute repository and the European EurOPDX resource, which has now established a panel of more than 1.500 PDX models for more than 30 pathologies [88].

#### 6.1.4. Limitations

The mouse Avatar has proved to be an invaluable model, fundamental for drug discovery, development of new drug combinations and biomarker studies, ultimately tailoring patient treatment. However, the latency period until tumor establishment and expansion in the mouse is a major constrain for the use of mPDXs to aid decision making for first clinical choices. Usually, there is a period of ~3–4 weeks since initial diagnosis until the start of treatment, and mPDXs take months to be established and expanded, not being compatible with the time frame needed for first clinical decisions. Consequently, mPDXs have been used for personalized medicine only in cases of relapsing/metastatic tumors. This is of extreme relevance, since postponing an effective treatment allows disease progression and ultimately tumor evolution and resistance, while patients are subjected to unnecessary toxicities. Also, the generation of an Avatar usually requires large amounts of fresh tumor material, being difficult to implant micro-biopsies in mice. Finally, the establishment of mPDXs is costly and resource-intensive, with limiting statistical power, not to mention the ethics implications of using an adult animal model. Thus, the zebrafish model is emerging as a complementary or alternative model.

### 6.2. Zebrafish Xenografts

Over the last years, the zebrafish has emerged as an in vivo model organism originally in the field of developmental biology, largely due to its external fertilization, rapid development and optical clarity [98,99,100]. The zebrafish genome is sequenced, sharing 70% of homology with humans, namely in crucial pathways involved in vertebrate development and cancer. It is also reported that 82% of disease-causing human proteins have an ortholog in zebrafish [101].

Several types of zebrafish xenografts can be generated: some where human tumor cells are implanted in very early embryos, like blastula embryos [102], others in larval stages (the most commonly used), and others in adult immune compromised zebrafish strains [103]. Similar to mPDXs, cells can be implanted in different sites, including the perivitelline space (PVS), yolk sac, duct of Cuvier, eye, brain ventricles or pericardial cavity [100]. Due to advantages discussed below, zebrafish xenografts could be considered an alternative to mice in complying with ethical standards such as the “3Rs” (replacement, reduction, refinement). In particular, using embryonic/larval stages provides high numbers of xenografts with a high statistical power and reduced ethical issues.

#### 6.2.1. Pioneers

In 2005, Lee et al. were forerunners in performing experiments with zebrafish xenografts by transplanting melanoma cells into blastula stage embryos [102]. One year later, Haldi and colleagues optimized parameters for xenotransplantation at the larval stage, including injection site, number of injected cells, injection method and larval incubation temperature [104]. This was the first study to show that human cancer cells injected in zebrafish larvae can proliferate, migrate and form masses in vivo [104]. This study was also the first to use 2 days post-fertilization (dpf) larvae as the host for injection, which became the standard protocol. Later in 2009, Marques et al. took a step forward and showed the possibility to follow invasion, circulation, migration and micrometastasis formation for both cancer cell lines and human primary cells, using transparent 2dpf larvae [105].

Since then, several zebrafish xenograft studies have been developed mostly with commercially available cell lines, derived from a wide range of cancer types, such as breast cancer, leukemia, lung cancer, CRC, pancreatic cancer, stomach cancer, glioblastoma, and melanoma (Table S1). This model has been successfully established to investigate several hallmarks of cancer, in particular angiogenesis [104,106,107,108,109,110,111,112], cancer cell invasion/extravasion [113,114,115], and micrometastasis formation [106,114,116] (Table S1). In addition, there are already several studies that use the zebrafish xenograft model in the process of drug discovery and pre-clinical evaluation of different types of therapies, such as chemotherapy, radiotherapy [117,118] and biological therapies [106] (Table S1).

#### 6.2.2. Advantages of the Zebrafish Larval Xenograft Model

**Figure 4:**
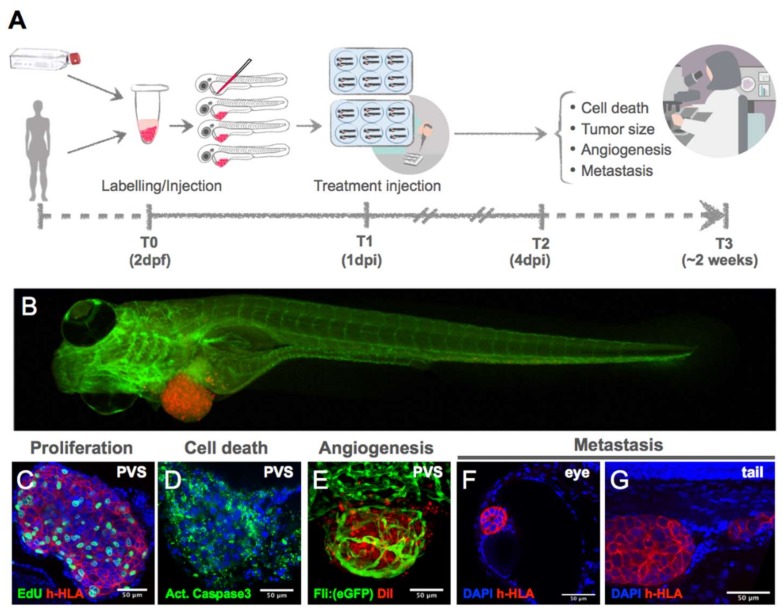
(**A**) Experimental setup for generating zebrafish xenografts. Cells derived from in vitro culture or primary human cells are labelled and microinjected in the PVS of 2dpf larvae. One day after injection, larvae are screened for successful injection and distributed in groups for testing chemo-, radio- and/or biological therapies. Three days after treatment, larvae (**B**) are fixed and processed for immunofluorescence for analysis of proliferation (**C**), cell death (**D**), angiogenesis (**E**), and metastatic potential (**F**,**G**). dpi: days post injection.

The zebrafish larval model presents numerous advantages, being the most important its speed—it can provide a one-week assay (Figure 4). This contrasts with mouse xenotransplants, which require months to generate a sufficient number of animals to perform studies [119]. This is essential for personalized medicine, since the test has to be performed in a time frame compatible with the first clinical decision.

The transparency of the embryos and existence of mutants without pigmentation, such as the casper line, offers the possibility to visualize tumor-associated processes, such as implantation, migration and micrometastasis formation [89,98,100,120,121,122]. One example was the work of Heilmann et al., where the authors used the transparent casper recipient for a quantitative measurement of the metastatic process and visualization of tumor cell extravasation at distant sites, one of the most difficult parts of the metastatic cascade to analyze in murine models [123]. Mouse models also allow for live imaging using multiphoton intravital imaging, but only at the tumor site [52,57]. In contrast, zebrafish xenografts allow for complete imaging of the whole animal and quantification of metastatic spread at a single-cell level [106,107,116].

In addition, the possibility of using transgenic embryos with GFP-labelled vasculature (*Tg:Fli1a*) [124], or mCherry-labelled macrophages (*Tg:Mpeg1*) [125] combined with xenotransplanted labelled tumor cells enables the real-time monitorization of tumor induced angiogenesis and tumor behavior, as well the observation of the tumor and host innate immune interactions [111,126].

Another advantage is the reduced number of tumor cells (~500 cells) necessary for successful transplantation. Since much less human material is required to generate a xenograft, establishing zPDXs using tumor biopsy samples can be more feasible than in mice. To the best of our knowledge, at least two studies already reported the generation of zPDX with biopsy samples [118,127]. Importantly, there is no need for cell culture amplification [105,106,118], thus reducing the time and number of artefactual steps required for in vitro adaptation.

The absence of the adaptive immune response until 8dpf [125] is also an important feature of the zebrafish larval model that allows xenograft engraftment. Therefore, human tumor cells usually are not rejected until that time point, avoiding the need of immunosuppressing agents or radiation, in contrast with murine models [128]. Other characteristics such as the small size; the ability to absorb compounds through the water, avoiding the burden of administering drugs to each animal individually (although is also possible to locally or systemically inject compounds that are not so easily absorbed); the reduced amounts of drugs required per test; the possibility to have a statistically-significant number of animals per assay; less ethics constrains and low costs of husbandry, make the zebrafish larvae a very attractive and promising in vivo model for human cancer studies.

#### 6.2.3. Zebrafish Patient-Derived (zPDX)-Avatars

Regarding zebrafish larval xenografts using patient-derived samples, few studies have been published so far (see Table S1). Nevertheless, these studies show promising results and the reliability of the zPDX model to establish different cancer types, as it is able to overcome some of the disadvantages of the murine PDXs such as the time required to develop an assay, as well as number of cells needed for implantation [106,115,128,129,130,131].

Pioneering work from Marques et al. [105] showed the possibility to transplant primary human tumors into zebrafish. Pancreas, colon and stomach primary tumors were transplanted either as tissue fragments or as cell suspensions into the yolk sac of the zebrafish larvae. Both types of samples showed invasive and metastatic behaviors, with cells disseminating through the whole zebrafish, suggesting that this is a good model to investigate tumor invasiveness and metastatic potential [105]. In 2016, Lin et al. showed that it is possible to generate zPDXs from multiple myeloma cells by injecting in the PVS rather than in the yolk sac, with engraftment rates of approximately 80% [112]. These authors were the first to show in a retrospective study that zPDXs can be a predictive tool for co-clinical assays. In this study, zPDXs were treated with bortezomib and lenalidomide, and showed a response equivalent to patient’s clinical outcome: sensitive tumors responded, whilst metastatic tumors did not, similarly to what was observed in patients (*n* = 6 patients) [112]. Subsequent work from Wu J.Q. et al. [129] showed that patient-derived gastric zPDXs were able to metastasize and recruit vessels into the tumor mass. A retrospective correlation with the patient clinical outcome was shown only for one patient.

As already mentioned, a major advantage of the zPDX model is its transparency, that allows single-cell resolution imaging without the need of tissue clearance methods. In 2017, Fior et al. [106] explored the strengths of the zebrafish larvae model, combined with high resolution microscopy techniques and with a robust battery of complementary analytic tools that validate the results obtained at the single-cell, molecular and histopathology levels. In this work the model was challenged and optimized in a systematic manner to study inter-tumor and intra-tumor heterogeneity. The effects of single mutations on tumor cell phenotype, angiogenesis and metastatic potential were also assessed, first using CRC cell lines, and then patient-derived samples. Cell proliferation was quantified using ki67 and mitotic indexes with single-cell resolution confocal images, in contrast to previous studies where cell proliferation was inferred from total cell numbers or tumor area, based on fluorescence measurements on stereoscope images. Also, with a 2h pulse of EdU, it was clear that human tumor cells were actively undergoing S phase, and therefore capable of proliferating in the PVS of zebrafish larvae at 34 °C. This validation of cell proliferation using several molecular markers was fundamental to demonstrate the full optimization of the zebrafish larvae xenograft model. Next, the possibility to screen the advanced CRC treatment guidelines from 1st to 3rd line of treatments in just 4 days was demonstrated. As readouts of treatment response, quantification of proliferation (mitotic index), apoptosis (activated Caspase 3 induction) and tumor size (n° of human nuclei) was performed. Another important step towards the validation of the zebrafish-larval model was the direct comparison between zebrafish and mouse xenografts, showing similar chemosensitivity profiles [106]. Finally, zPDX Avatars were generated and a retrospective study was performed, showing that zPDX Avatars could predict patient clinical outcome in 4 out of 5 patients (80%). Furthermore, cetuximab showed no effect on 3 additional zPDXs Avatars. These results were later correlated with the presence of KRAS or BRAF mutations, which are described to confer resistance to cetuximab treatment [106].

Just like with chemotherapy, there are tumors that do not respond to radiotherapy. To address this, zPDX-Avatars from rectal cancer biopsies were recently used to predict patient clinical outcome in two case studies, in a neoadjuvant chemoradiotherapy setting [118].

An alternative approach to direct injection of patient cells is to expand tumor cells in vitro before injection, with the possibility of introducing genetic modifications. This approach was recently described by Wang et al. [132], where in addition to the dual labelling of tumor cells and fibroblasts with fluorescent reporters, the anti-apoptotic gene BCL2L1 was also overexpressed to prolong the life span of the xenografted cells. The zebrafish used were also humanized to express insulin and insulin growth factor-1 to better support survival and proliferation of implanted human cells. Although these authors demonstrated a dose-dependent response to drug treatments, no correlation with patient clinical outcomes was performed.

#### 6.2.4. Adult Zebrafish Xenografts

Recently, another major breakthrough was achieved in the field, with the possibility to efficiently engraft human patient-derived tumor cells into adult immunodeficient zebrafish [103]. To this end, the authors generated optically clear zebrafish mutants for protein kinase DNA-activated catalytic polypeptide (*prkdc*) and interleukin 2 receptor gamma (il2rg), that lack T, B, and NK cells. The authors show that these transgenic adult zebrafish robustly engraft human cancer cells at 37 °C, with a growth kinetics similar to the one observed in mPDXs.

This approach allows the study of cell proliferation and migration at a single-cell level in the whole adult fish, contrasting with the limitations of intravital imaging done in mPDX. Cells were derived from one patient with glioblastoma, two patients with embryonal rhabdomyosacoma (RMS), two patients with metastatic BRAF(V600E)-induced melanomas and one patient with therapy resistant lobular breast cancer. Prior to zPDX generation, stable cell lines were expanded and generated by lentiviral transduction and either passaged in vitro or obtained from previously generated mPDXs. An innovation from these authors, besides the double mutant, was the adaptation of the rearing conditions to 37 °C and injection of cells mixed with l-Clodronate and matrigel, to avoid tumor clearance by macrophages. Drug treatments (olaparib and temozolomide) were administered by oral gavage and conducted for 28 days, after which zebrafish were examined histologically or by confocal microscopy. The obtained results suggest the combination of olaparib and temozolomide as a potential treatment combination for RMS. The results further show that this drug combination leads to G2-cell-cycle arrest, already observed at 2 days post-treatment, suggesting that G2 arrest precedes apoptosis [103]. However, this study did not yet generate zPDXs directly from patient cells (cells were first expanded in vitro or derived from mPDXs), nor did it demonstrate a correlation with the patient clinical outcome, but new exciting studies are probably coming soon.

#### 6.2.5. Disadvantages

##### Not a Mammal...

One of the major drawbacks of the zebrafish xenografts, also shared with mouse PDX, is that engraftment efficiencies vary significantly, not allowing the successful establishment of a zPDX from every single sample available. This may happen due to innate immune rejection of the inoculated tumor cells or bad quality of the samples (necrosis). To overcome this, some chemicals and irradiation have been employed in certain studies to act as immunosuppressors [100]. However, these can ultimately lead to misleading results.

Another critique of the model is its incubation temperature. Since zebrafish are typically reared at 28–29 °C, and human cells thrive at 37 °C, the compromise usually adopted is to raise zebrafish xenografts at 33–35 °C, which can have an impact on the physiology of the fish and the biology of the tumor cells [128]. Lastly, another limitation of the zebrafish could be the pharmacokinetics and pharmacodynamics of some drugs. Although it has been demonstrated that larvae have the ability to perform metabolic reactions, and that drug distribution, metabolism, and excretion are similar to humans, these fields are still scarcely explored in zebrafish [100,133].

#### 6.2.6. Zebrafish Avatars: Standardize Methods and Increase Retrospective and Prospective Studies

In the last years, some aspects of the zebrafish-larvae xenograft technique have been established and standardized such as injection in the larval stage at 2dpf. There are, however, other features that are variable between research groups, like the site of injection. Similarly to mPDX, the best site of injection is still under debate. While the majority of authors inject subcutaneously in the PVS or in the yolk sac of zebrafish larvae, others inject orthotopically, for example in the brain to study glioblastoma [134]. Many times the yolk sac is preferred, as it is bigger and easier to inject and can accommodate more cells. However, it has the disadvantage of being an environment composed of bulk proteins or lipids [135], which might impact tumor phenotype and increase cell death. Alternatively, subcutaneous injection in the PVS seems to create more favorable conditions for tumor mass formation, cell proliferation, angiogenesis and metastization [106,109,118].

The temperature of zPDX incubation is perhaps the most controversial issue. The range goes from 28 to 37 °C, being 34 °C the most commonly used in published works (Table S1). Although the ideal temperature for human tumor cell proliferation is 37 °C, zebrafish larvae do not develop correctly at this temperature, developing cardiac problems and edema [136] that compromise zPDX survival. At 34 °C, the physiology of the fish is less compromised and tumor cells were shown to actively proliferate [106].

The readout harmonization is another critical step for zPDX-Avatar validation. Most published works analyze cell death or proliferation with one assay only and using unspecific methods like measuring tumor volume from fluorescence intensity. Lipophilic dyes, although very useful, might not be very reliable. Some cells stain better than others, and dead cells or debris can be retained in the yolk sac, leading to false signals. For example, phagocytic cells such as macrophages can become stained after “eating” these debris, leading to false positive signals, including micrometastasis.

Therefore, for a careful and thorough analysis, it is indispensable that a set of analytic tools should be used for validation of the results. Direct assays of cell functionality and death, such as immunofluorescence for activated Caspase 3 for apoptosis, and unequivocal detection of human cells (anti-human specific antibodies) should be performed. Although confocal microscopy and analysis is laborious and time consuming, and therefore is not compatible with high throughput, it is our opinion that all steps must first be validated, and all caveats learned, before moving onto more automated methods. Nevertheless, additional readouts of other types of cell death (like necrosis, autophagy, etc.) or cell senescence should be developed, to increase the amount of information and accuracy of results.

In summary, to improve the robustness and reproducibility of the larvae xenograft model, we propose that protocols should be harmonized between labs, at a set of parameters (Figure 4):

- Injection site: PVS.
- Temperature: 34 °C.
- N° of cells: >500 cells/xenograft.
- Scoring of injection efficiency: discard badly injected fish and sort by size.
- Confocal imaging of analytical tools (readouts), such as:
  ◦ Proliferation (quantification of mitotic figures with DAPI or pHH3);
  ◦ Tumor size (DAPI counting);
  ◦ Apoptosis with activated Caspase3 antibodies or equivalent;
  ◦ Development of new readouts for cell death;
  ◦ Unambiguously detection of human cells (such as anti-human HLA, anti-human mitochondria or anti-human nuclei antibodies).

Importantly, although many advances have been made, additional retrospective studies with many more patients are needed for robust model validation as a drug screening tool for personalized medicine applications. Also, as this model gains strength, we hope that prospective studies on individual patients will emerge to help clinicians tailor the treatments they provide to their patients.

### 6.3. Drosophila Avatars—A Genetically Engineered Model

Alternatively to PDXs, genetically engineered models can also be used as Avatars. Very recently, Bangi et al. [137] used the fruit fly (*Drosophila melanogaster*) to create genetically engineered fly Avatars, which had the same mutations (oncogenes and tumor suppressors) as the patient’s cancer (9 different mutations). This was a patient with terminal colon cancer who developed resistance to many therapies. Approximately 300,000 fly Avatars were developed with the corresponding mutations in the drosophila gut. With a robotic system, the genetically engineered flies were used to screen a panel of 121 FDA-approved drugs to narrow-down which compounds could be more efficient for that particular patient. Trametinib together with zoledronate showed the most effective action and were applied to the patient. The combination resulted in the shrinkage of the patient’s tumor, but after 11 months the patient developed resistance [137].

This proof of concept study exemplifies how an in vivo high throughput model can be so powerful. Although 9 driver mutations are very impressive, these do not represent the heterogeneity of the original tumor, such as epigenetic alterations and interactions with the TME, and patient cells are not directly tested.

## 7. No Model Fits All

**Figure 5:**
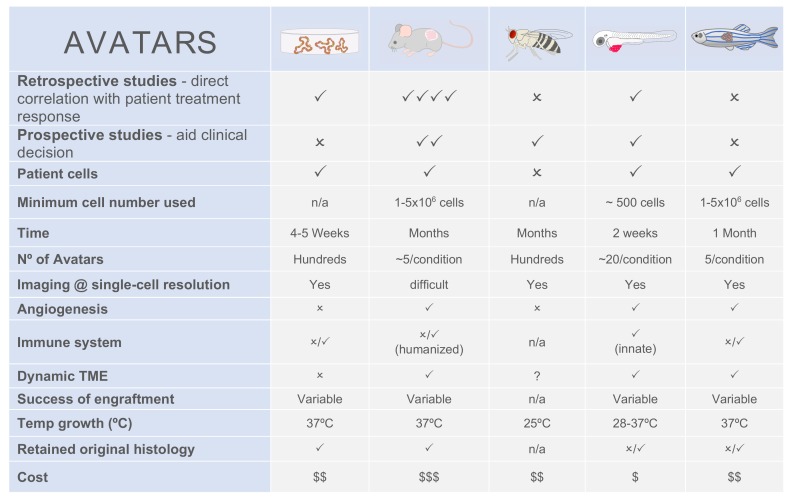
Comparison between patient-derived Avatar models.

Overall, all models have their pros and cons (Figure 5) and no perfect model exists, except the patient himself. That is exactly what we, as scientists, are trying to avoid by developing Avatar models. Since each model answers specific biological questions, a step forward could be the use of multi-model systems as complementary tools, being aware of the strengths and limitations of each one. For instance, Avatar models that need more time to develop could be used to advise metastatic patients and follow tumor evolution, whereas fast Avatar models would be used for first line therapies

## 8. The Future—Combination of Precision and Personalized Approaches

Technology is advancing at an uncontrolled speed and the era of *“omics”*, big data and artificial intelligence has generated enormous amounts of information that led to the successful development of many targeted approaches to cancer treatment [138]. System biology approaches or mathematical oncology feed on these massive data and are of great promise [139]. However, understanding cancer biology is still far from being achieved.

Precision medicine approaches, such as pharmacogenomics, have proven their great value on patient stratification based in the genomic information of patients and how this affects drug response [13]. Amongst the many options available, this methodology has allowed clinicians to narrow down treatment options, reducing the number of trial and error attempts. However, this method does not take into consideration the in vivo cellular response upon drug challenge. More specifically, it does not take into account all the possible interactions that may occur, such as genetic and protein–protein interactions, clonal selection, intracellular and metabolic rewiring mechanisms, tumor-immune and tumor-stroma interactions. These and other variables are responsible for the lack of drug sensitivity or for the acquisition of resistance mechanisms [23].

**Figure 6:**
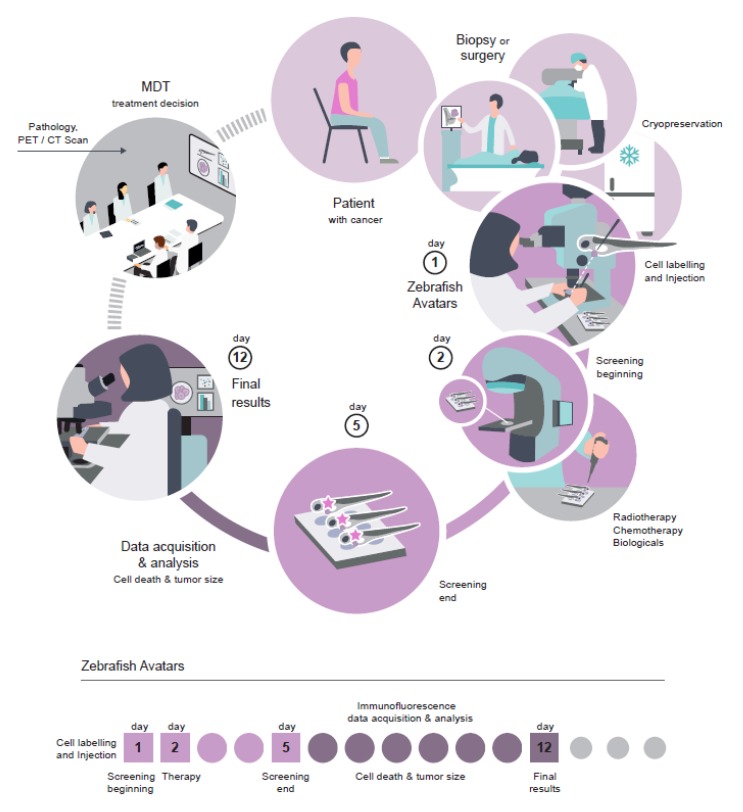
Workflow of zebrafish Avatars in the context of personalized medicine.

Patient-derived Avatars are being developed both in vitro and in vivo with the aim of directly challenging tumor cells and avoid trial and error approaches, so often seen in the clinic. Zebrafish Avatars are gaining attention due to the low number of cells needed for zPDX generation, the optical clearness of the zebrafish and, above all, the short time needed for therapy profiling [106]. The zPDX Avatars do not require tumor cell expansion and the results (cell death, angiogenesis, and micrometastasis detection) can be obtained in just 4 days, which is compatible with the time frame needed for clinical decisions (Figure 6). The difference in time scale of the assay is not due to the zebrafish’s biology being faster, but rather because zebrafish larvae are 10,000 times smaller than adult mice. This allows the injection of less cells per animal (~500 cells in zebrafish instead of 1 × 10^6^ in the mouse—2000 times more material needed in mice) and, therefore, the attainment of a higher number of xenografts for statistical analysis. Most importantly, this reduction of scale allows resolution and visualization of drug response, angiogenesis and formation of micrometastasis at the single-cell level [106].

Although precision oncology and personalized medicine are often considered synonyms, they are focused on different aspects of the same goal. Precision oncology focuses mainly on correlating specific molecules (genes, metabolites etc.) with effective drug treatments, leading to the discovery of new biomarkers that allow the reduction of treatment options [13]. It does not, however, consider each individual patient. In personalized medicine, on the contrary, patient-derived live cells are challenged with specific drugs, and responses can be evaluated in real time and directly transposed to the clinic [128]. However, not all possible therapeutic options can be tested in Avatars due to the restricted amount of patient material. The solution is to take advantage of the two approaches: precision oncology will reduce treatment options and then personalized tests on Avatars will determine the best option. We believe that by combining these two approaches, efficacy rates will increase and patients will not be subjected to unnecessary treatments, improving their quality of life and reducing healthcare costs.

## Supplementary Materials

The following are available online at https://www.mdpi.com/2073-4409/9/2/293/s1, Table S1: Selection of zebrafish xenografts papers; in blue: papers where patient-derived cells were used and in grey papers where cell lines were used.

## Abbreviations

EdU	5-Ethynyl-2′-deoxyuridine
Bcl-2	B-cell lymphoma 2
CSRAs	Cell culture chemotherapy sensitive and resistant assays
CDXs	Cell-Derived-Xenografts
CRC	Colorectal cancer
dpf	Days post-fertilization
DBP	Dynamic BH3 profiling
EGFR	Epidermal growth factor receptor
ER	Estrogen receptor
ECM	Extracellular matrix
ESMO	European Society for Medical Oncology
FDA	Food and Drug Administration
HER2	Herceptin-2
HLA	Human leukocyte antigen
MOMP	Mitochondrial Outer Membrane Permeabilization
mPDX	Mouse Patient-Derived Xenografts
NCCN	National Comprehensive Cancer Network
PDXs	Patient-Derived Xenografts
PVS	Perivitelline space
pHH3	Phospho-histone H3
PARP	Poly ADP ribose polymerase
PD-1	Programmed cell death-1
prkdc	Protein kinase DNA-activated catalytic polypeptide
RMS	Rhabdomyosacoma
TME	Tumor microenvironment
zPDX	Zebrafish Patient-Derived Xenografts
