# Supplementary material for: Zebrafish Avatars towards Personalized Medicine—A Comparative Review between Avatar Models
Source: Cells. 2020 Jan 25;9(2):293. doi: 10.3390/cells9020293 (PMC7072137; doi:10.3390/cells9020293)
Supplement: Supplementary file 1 [file cells-09-00293-s001.pdf]

Article	Cancer type	Goals of the study	Cell lines	Patient xenografts	Treatment correlation	Genetic correlation	Readouts	Stage	T (ºC)
Yan et al, 2019.	Rhabdomyosarcoma<br>Melanoma<br>Breast cancer<br>Glioblastoma	Zebrafish reared at 37 °C improve engraftment; drug response.	ERMS RD, ARMS Rh41<br>UACC62<br>MDA-MB-231	6 patients (cultured)	No	No	• Live cell tracking;<br>• Tumor growth (fluoresce. intensity);<br>• H&E;<br>• Proliferation (Ki67);<br>• Apoptosis (TUNEL).<br>Confocal microscopy	Adult	37
Wang et al, 2019.	Pancreatic cancer	Optimization of zPDX as a screening platform for clinical use.	-	1 patient (cultured)	No	No	Fluorescence intensity (stereoscope)	Larvae	32
Lin et al, 2019.	Hepatocellular carcinoma	Tumor formation, proliferation, migration, angiogenesis	293T/EDN1	-	No	No	• Tumor growth (fluoresce. intensity);<br>• Angiogenesis;<br>• Live imaging;<br>Fluorescent microscopy.	Larvae	28-37 (gradient)
Ji et al, 2018.	Acute myeloid leukemia	Anti-tumor effects of the phytotoxin stemphol	U-937	4 patients (cultured)	No	No	• Tumor size (fluoresce. intensity).<br>Fluorescent microscopy.	Larvae	28.5
Wu et al, 2017.	Gastric cancer	Proliferation, angiogenic and metastatic activities, drug response	AGS,<br>SGC-7901	9 patients (cultured)	Yes<br>(1 out of 1)	No	• Cell viability (CCK8 kit);<br>• Nuclear stain (DRAQ5);<br>• Angiogenesis;<br>• H&E;<br>• Cell number (tumor dissociation).<br>Fluorescent and confocal microscopy.	Larvae	32
Leung et al, 2017.	NSCLC	Silencing of PAPSS1 sensitizes NSCLC cells to cisplatin treatment	A549	-	No	No	• Cell number (by tumor dissociation)<br>Fluorescent *microscopy*.	Larvae	35
Canella et al, 2017.	Glioblastoma	Onalespib in combination with temozolomide as a therapeutic approach against gliomas.	U251HF	-	No	No	• Live imaging;<br>• % xenografts survival;<br>• Tumor growth (automatic quantification).<br>Confocal microscopy	Larvae	32
Gaudenzi et al, 2017.	Neuroendocrine tumors	Tumor-induced angiogenesis and cell invasiveness	--	8 patients (cultured)	No	No	• Angiogenesis;<br>• H&E;<br>• Migration.<br>Fluorescent and confocal microscopy.	Larvae	32

Article	Cancer type	Goals of the study	Cell lines	Patient xenografts	Treatment correlation	Genetic correlation	Readouts	Stage	T (ºC)
Fior et al, 2017.	Colorectal cancer	Tumor proliferation, metastatic and angiogenic potentials; drug response.	SW480, SW620, HCT116, HT29, Hke3	10 patients (direct)	Yes (4 out of 5)	Yes (3 out of 3)	• Tumor size (number of cells);<br>• % apoptosis;<br>• Angiogenesis;<br>• % mitosis;<br>• % metastasis;<br>• H&E;<br>• Live imaging;<br>Confocal microscopy.	Larvae	34
Roh-Johnson, 2017.	Melanoma	Role of immune cells in tumor cell motility in vivo.	A375P A375M1, WM266-4, B16F10, 1205Lu, WM793, Mel-624; Zmel (zebrafish melanoma cells).	-	No	No	• Live imaging;<br>• Tumor cell dissemination;<br>• % macrophages;<br>• Time quantification of macrophage/tumor cell contact;<br>• Whole-mount IF.<br>Fluorescent and confocal microscopy.	Larvae	31
Hung et al, 2016.	Breast cancer	Effect of visfatin on migration and invasion.	MDA-MB-231	-	No	No	• Cell invasion (dissemination of labeled cells).<br>Fluorescent microscopy.	Larvae	32.5
Mercatali et al, 2016.	Breast cancer	Tumor behavior.	MDA-MB-231 MCF-7	Primary cells from bone metastasis, cultured (1 patient)	No	No	• Metastasis.<br>Fluorescent microscopy.	Larvae	34
Ghotra et al, 2015.	Prostate cancer	Role of SYK (spleen kinase) as a potential new drug target.	PC3, DU145, LNCaP, C4-2B	-	No	No	• Tumor cell spreading;<br>• Cumulative distance of cells per embryo;<br>• Mean cumulative distance (automatic quantification).<br>Confocal microscopy.	Larvae	34
Chen et al, 2015.	Retinoblastoma	Tumor invasion and metastasis.	SJmRBL-8 (mouse) RB355, WERI-Rb1	-	No	No	• Tumor areas and disseminated tumor cell (automatic quantification);<br>• % zebrafish with metastasis;<br>• Averages of maximal distance of metastatic foci.<br>Fluorescent and confocal microscopy.	Larvae	28.5

Article	Cancer type	Goals of the study	Cell lines	Patient xenografts	Treatment correlation	Genetic correlation	Readouts	Stage	T (ºC)
**Bentley et al, 2015.**	**T-cell acute lymphoblastic leukemia**	**Drug response.**	**Jurkat, Karpas45, TALL1**	**2 patients (cultured 12h) (biopsy bone marrow)**	**No**	**Yes (2 out of 2)**	**• Number tumor cells (dissociation PML + cells<br>Fluorescent and confocal microscopy.**	**Larvae**	**35**
Chapman et al, 2014.	Melanoma	Invasion properties and cooperation of tumor cells in a heterogeneous setting	UACC62, WM266-4	-	No	No	• Quantification of invasion;<br>• Whole-mount IF.<br>Confocal microscopy.	Larvae	34
Ban et al, 2014.	Ewing sarcoma	Inhibition of SIRT1 interferes with tumor growth and migration in vivo.	TC252, A673	-	No	No	• Proliferation (tumor growth) and distance of migration based on mCherry labeled foci.<br>Confocal microscopy.	Larvae	34
Van der Ent et al, 2014.	Ewing sarcoma	Proliferation, migration, and angiogenesis.	CADO-ES, EW3, EW7, L1062, TC32, TC71, SK-N-MC	-	No	No	• Whole-mount IHC;<br>• H&E;<br>• Angiogenesis;<br>• Migration;<br>• Interaction with immune system;<br>• Tumor burden (mCherry area).<br>Confocal microscopy.	Larvae and juvenile (35dpf)	34
He et al, 2012.	Prostate cancer<br>Breast cancer	Tumor cell invasion, tumor vascularization and micrometastasis formation.	MAE, FGF-T-MAE, 4T1 (mouse)<br>ZF4/PAC2 (zebrafish)<br>PC3, MDA-MB-231	-	No	No	• Live imaging;<br>• Whole-mount IHC;<br>• Angiogenesis;<br>• % metastasis;<br>• Microangiography;<br>• Proliferation (pH3);<br>• Immune system interaction;<br>• Neutrophils migration.<br>Confocal /2-Photon microscopy	Larvae	34
Stoletov et al, 2010.	Fibrosarcoma<br>Breast cancer<br>Colorectal cancer	Behaviour of metastatic human cancer cells undergoing extravasation.	HT1080, SW620, SW480, MDA-MB-435, MDA-MB-231,	-	No	No	• Cell extravasation;<br>• Intravascular migration (live imag.);<br>• Whole-mount IF;<br>• Blood vessel wall integrity-dextran.<br>Confocal microscopy.	Larvae	35.5

Article	Cancer type	Goals of the study	Cell lines	Patient xenografts	Treatment correlation	Genetic correlation	Readouts	Stage	T (ºC)
**Marques et al, 2009.**	**Pancreatic cancer<br>Colon cancer<br>Stomach cancer**	**Analysis of metastatic behaviour of human tumor cells.**	**EpRas (mouse)<br>PaTu8988-S,<br>PaTu8988-T**	**7 patients (direct)**	**No**	**No**	**• Whole-mount IF;<br>• H&E;<br>• Cell invasion and metastasis.<br>Confocal microscopy.**	**Larvae**	**35**
**Weiss et al, 2009.**	**Pancreatic cancer**	**MicroRNA-10a—required and sufficient for tissue invasion and metastasis of pancreatic tumor cells.**	**PaTu8988T/S, AsPC1, Capan1/2, MiaPaCa2, PANC1, PaTu8902**	**3 patients (cultured)**	**No**	**No**	**• Metastasis;<br>• H&E.<br>Fluorescent microscopy.**	**Larvae**	**35**
Lee et al, 2009.	Ovarian cancer<br>Breast cancer	Tumor-induced angiogenesis, invasion and metastasis.	T241 and LLC (mouse) OVCAR 8 MDA-MB-231	-	No	No	• Angiogenesis (vessel density);<br>• Tumor volume;<br>• Nº of disseminated cells;<br>• Metastasis maximal distance.<br>Confocal microscopy.	Larvae	28
Nicoli et al, 2007.	Ovarian<br>Breast	Tumor-induced angiogenesis.	MAE, FGF2-T-MAE and B16-BL16 (mouse) Tet-FGF2; A2780, MDA-MB-435	-	No	No	• Whole-mount in situ hybridization;<br>• Angiogenesis;<br>• DAPI-stained transverse sections;<br>• Neo-vessel quantification.<br>Fluorescent microscopy.	Larvae	28
Haldi et al, 2006.	Melanoma<br>Colorectal<br>Pancreatic	Optimization of xenotransplantation; proliferation, migration and mass formation.	WM-266-4<br>SW620<br>FG CAS/Crk<br>CCD-1092Sk	-	No	No	• Single cell dissociation (DiI);<br>• Angiogenesis;<br>• Whole-mount IHC;<br>• Whole-mount IC;<br>• Fluorescent/2-photon microscopy.	Larvae	35

In Blue – patient-derived cells were used.
